# Genetic landscape of interval and screen detected breast cancer

**DOI:** 10.1038/s41698-024-00618-6

**Published:** 2024-05-28

**Authors:** Charlie Mills, Amit Sud, Andrew Everall, Daniel Chubb, Samuel E. D. Lawrence, Ben Kinnersley, Alex J. Cornish, Robert Bentham, Richard S. Houlston

**Affiliations:** 1 Division of Genetics and Epidemiology, The Institute of Cancer Research, Sutton, Surrey, SM2 5NG UK; 2 Department of Medical Oncology, Dana-Farber Cancer Institute, Boston, MA USA; 3 Harvard Medical School, Boston, MA USA; 4 Broad Institute of MIT and Harvard, Cambridge, MA USA; 5 Centre of Immuno-Oncology, Nuffield Department of Medicine, University of Oxford, Oxford, UK; 6 University College London Cancer Institute, University College London, London, UK

**Keywords:** Breast cancer, Cancer genetics, Cancer screening

## Abstract

Interval breast cancers (IBCs) are cancers diagnosed between screening episodes. Understanding the biological differences between IBCs and screen-detected breast-cancers (SDBCs) has the potential to improve mammographic screening and patient management. We analysed and compared the genomic landscape of 288 IBCs and 473 SDBCs by whole genome sequencing of paired tumour-normal patient samples collected as part of the UK 100,000 Genomes Project. Compared to SDBCs, IBCs were more likely to be lobular, higher grade, and triple negative. A more aggressive clinical phenotype was reflected in IBCs displaying features of genomic instability including a higher mutation rate and number of chromosomal structural abnormalities, defective homologous recombination and *TP53* mutations. We did not however, find evidence to indicate that IBCs are associated with a significantly different immune response. While IBCs do not represent a unique molecular class of invasive breast cancer they exhibit a more aggressive phenotype, which is likely to be a consequence of the timing of tumour initiation. This information is relevant both with respect to treatment as well as informing the screening interval for mammography.

## Introduction

The high survival rates associated with the early detection of localised breast cancer together with the screening properties of mammography, have provided the rationale for population breast cancer screening programs^1–3^. In the UK, the National Health Service (NHS) invites women aged between 50 and 70 for 3-yearly mammography, but with provision for those aged 45 and over to be screened if self-referred. Although studies suggest a mammography every 2–3 years may reduce breast cancer-specific mortality by a fifth^4^ around 30% of breast cancers in women attending screening are interval breast cancers (IBCs) diagnosed between screening episodes^5^.

While technical and patient factors may in part explain the incidence of IBCs^6,7^, studies have suggested IBCs may represent a more aggressive form of breast cancer^8^. This would accord with the observation that most IBCs are not visible on the index screen in retrospective review^8^. Exploring possible genetic differences between IBCs and screen-detected breast cancers (SDBCs) has the potential to inform mammographic screening and patient management. Previous analyses of IBCs have so far predominantly focused on specific genes and studies have typically been based on small patient numbers^9,10^.

**Fig. 1:**
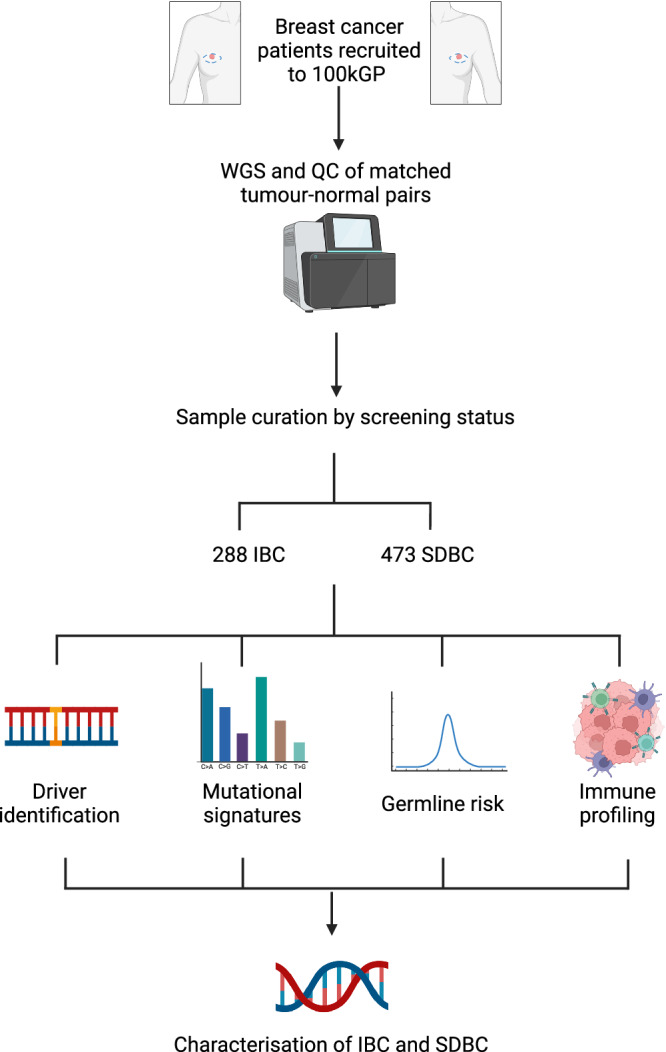
Study overview. WGS whole genome sequencing. Created with BioRender.

To provide the most comprehensive analysis of the genomic landscape of IBC, we analysed whole genome sequencing (WGS) data generated on 288 IBCs recruited to the UK’s 100,000 Genomes Project (100kGP). The results of our study highlight distinct clinical, genetic and evolutionary differences between IBC and SDBC (Fig. 1).

## Results

### Clinico-pathological differences

After curation we based our analysis on 288 IBCs and 473 SDBCs. The clinical details of the 761 cases are summarised in Table 1. Overall, 72% (*n* = 548) of the cases were self-reported to be White British and there was no difference in the frequency of ethnicities between IBCs and SDBCs. There was no significant difference in the tumour purity of IBCs and SDBCs (*P* = 0.17), making it unlikely that study findings will have been affected by sample collection and processing. Interval breast cancers were more likely to be larger tumours than SDBCs (median size 24 mm and 20 mm respectively, *P* = 1.9 ×10^−5^), be lobular (OR = 1.61, 95% confidence interval (CI) 1.07–2.42, *P* = 0.02), be higher grade tumours (OR = 1.83, 95% CI 1.45–2.31, *P* = 3.8 ×10^−7^), be oestrogen receptor (ER) negative (OR = 0.43, 95% CI 0.26–0.73, *P* = 1.11 ×10^−3^), be progesterone receptor (PR) negative (OR = 0.40, 95% CI 0.25–0.65, *P* = 4.45 ×10^−5^), be TN (OR = 2.01, 95% CI: 1.02–3.98, *P* = 0.03) and patients were more likely to be diagnosed with nodal disease at presentation (OR = 1.56, 95% CI: 1.12–2.17, *P* = 7.13 ×10^−3^) (Table 1).

**Table 1:** Clinical characteristics of IBCs and SDBCs

		Interval breast cancer	Screen detected breast cancer	*P*-value, Odds ratio (95% CI)
Total		288	473	
Histology	Ductal	230 (79.9%)	409 (86.5%)	0.019, 0.62 (0.41, 0.94)
	Lobular	58 (20.1%)	64 (13.5%)	
Age	Median (years)	60	60	0.85
	Range (years)	47–70	47–70	
Tumour size	Median (mm)	24	20	1.9 × 10^−5^
	Range (mm)	9–95	3.5–115	
Laterality	Right	136 (47.2%)	245 (51.8%)	0.23, 0.83 (0.61, 1.13)
	Left	152 (52.8%)	228 (48.2%)	
Grade	G1	20 (6.9%)	64 (13.5%)	3.8 × 10^−7^, 1.92 (1.51–2.47)
	G2	140 (48.6%)	284 (60.0%)	
	G3	127 (44.1%)	123 (26.0%)	
	GX	<5	<5	
ER status	Positive	193 (67.0%)	356 (75.3%)	1.11 × 10^−3^, 0.43 (0.26, 0.73)
	Negative	40 (13.9%)	32 (6.8%)	
	U	55 (19.1%)	85 (17.9%)	
PR status	Positive	106 (36.8%)	222 (46.9%)	4.45 × 10^−5^, 0.40 (0.25, 0.63)
	Negative	60 (20.8%)	49 (10.4%)	
	U	122 (42.4%)	202 (42.7%)	
HER2 status	Positive	26 (9.0%)	32 (6.8%)	0.20, 1.44 (0.80, 2.57)
	Borderline	26 (9.0%)	55 (11.6%)	
	Negative	211 (73.3%)	366 (77.4%)	
	U	25 (8.7%)	20 (4.2%)	
Nodal status	Positive	110 (38.2%)	136 (28.8%)	7.13 × 10^−3^
	Negative	157 (54.5%)	303 (64.0%)	
	U	21 (7.3%)	34 (7.2%)	
Triple Negative		22 (7.6%)	20 (4.2%)	0.03, 2.01 (1.02, 3.98)

*P*-values were evaluated using a t-test for age and tumour size, a Cochran-Armitage trend test for grade, and Fisher’s exact test for all other variables.

*U* unknown status.

Patients with lobular IBCs were more likely to be older at diagnosis than those with SDBCs (mean ages 61.1 years and 58.5 years respectively; *P* = 0.03). While patients with ductal TN IBCs were older than those with SDBCs (mean age 61.1 years and 56.6 years respectively; *P* = 0.03), patients with ductal ER-positive breast IBCs cancers tended to be younger than those with SDBC with the same histology (mean ages 58.2 years and 59.8 years respectively; *P* = 0.02).

### Germline variation and polygenic scores

We identified 17 patients with IBCs who were carriers of a pathogenic mutation in one of the HBOC genes (13 *BRCA2*, 2 *CHEK2*, 1 *MSH6* and 1 *BRCA1*) and 27 patients with SDBC who were HBOC gene carriers (12 *BRCA2*, 7 *ATM*, 3 *CHEK2*, 1 *BARD1*, 1 *BRIP1*, 1 *BRCA1*, 1 *MSH6*, 1 *PALB2*) (Supplementary Data 1). The frequency of patients with *BRCA*-mutations was marginally, but not statistically significantly, higher in IBCs compared to SBDCs (OR = 1.80, 95% 0.77-4.23, *P* = 0.16). Considering all HBOC genes, there was also no significant difference in carrier frequency (5.9% versus 5.7%).

We generated PGS on a per sample basis to investigate the relationship between genetically predicted BMI and breast density as well as modifiable breast cancer risk factors in IBC and SDBC. In a case-only analysis, while SDBCs had a higher PGS for overall breast cancer than IBC, this was not significant (*P* = 0.68). Similarly, there was no difference in genetically predicted breast density or BMI between IBCs and SDBCs (*P*-values 0.09 and 0.65 respectively; Supplementary Data 2).

### Somatic alterations

**Fig. 2:**
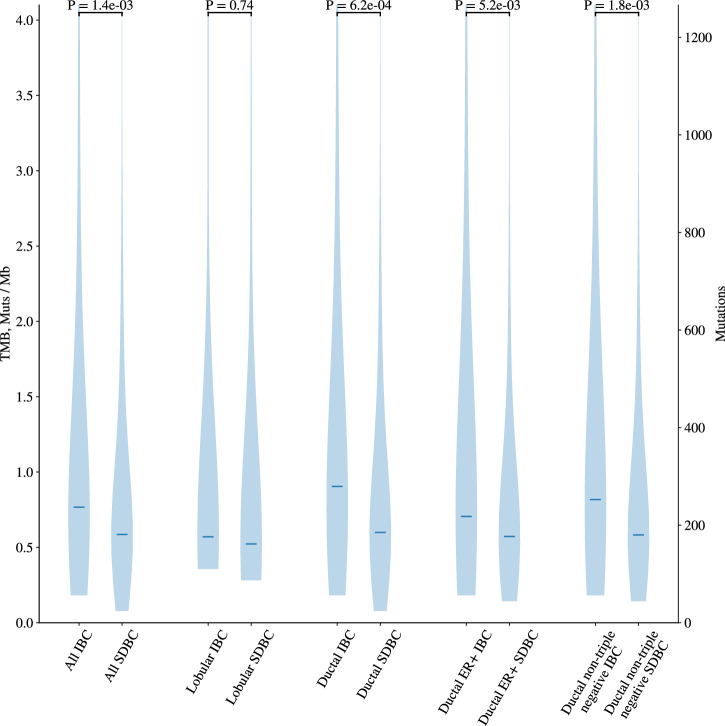
Mutational burden in IBC and SDBC. The mutational burden distribution for IBCs and SDBCs in selected cohorts. The median of the distribution is indicated by the horizontal line and *P*-values calculated using a Wilcoxon rank sum test.

The frequency of somatic mutations (single nucleotide variants and indels) was significantly higher in all IBCs compared to all SDBCs (1.47/Mb (0.18–18.55) and 1.06/Mb (0.08–19.05) respectively, *P* = 1.43 ×10^−3^). This significant difference was also shown when stratified by tumour subtype - all ductal (*P* = 6.32 ×10^−4^), ductal ER-positive (*P* = 5.23 ×10^−3^) and ductal non-TN tumours (*P* = 1.81 ×10^−3^) (Fig. 2, Supplementary Fig. 1). After adjustment for tumour grade the mutational burden remained significantly higher in all IBCs compared to all SDBCs (*P* = 0.03).

**Fig. 3:**
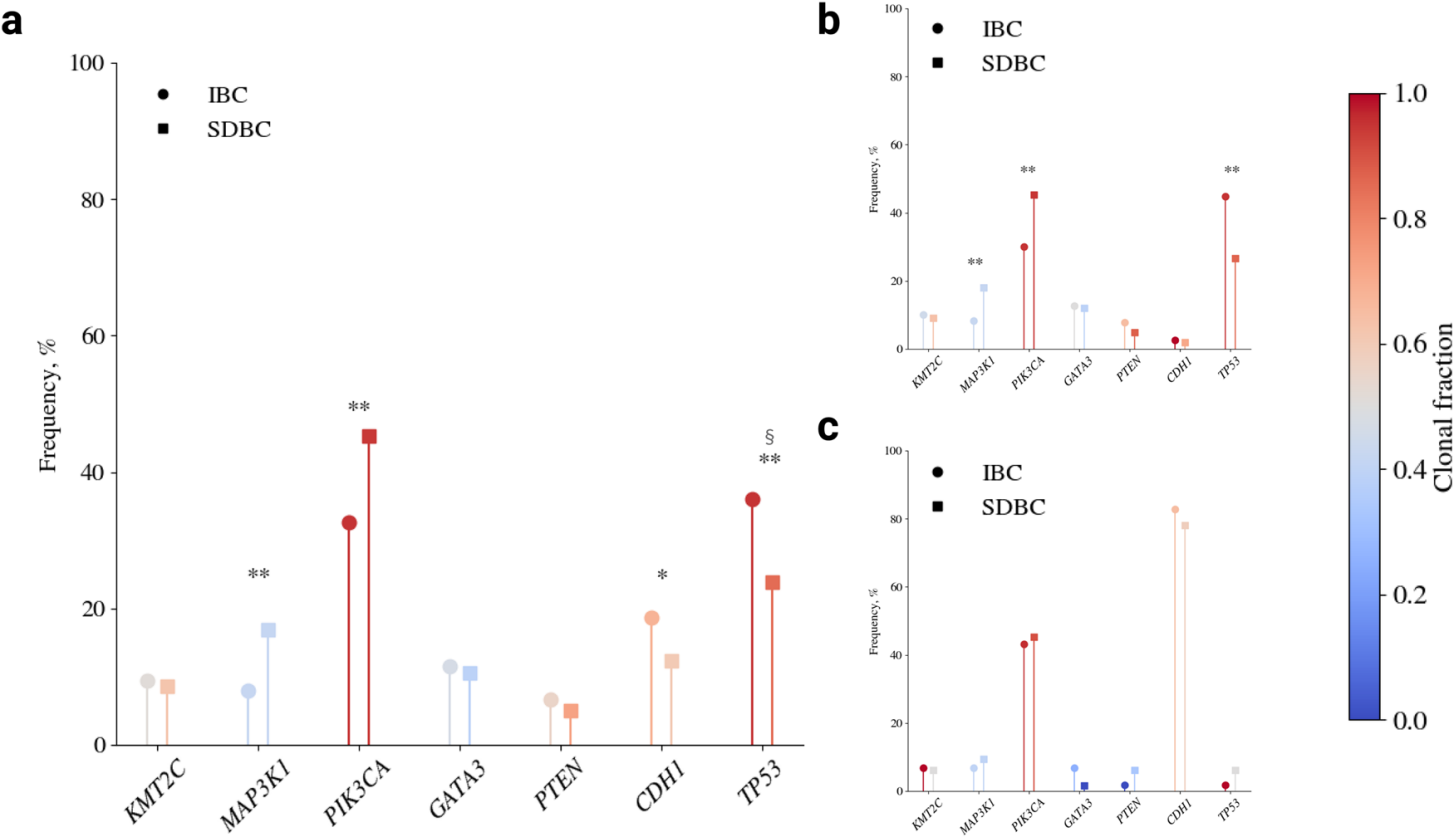
Frequency and clonality of driver gene mutations in IBC and SDBC. The mutational frequency of driver genes for: **a** all IBCs and SDBCs; **b** ductal IBCs and SDBCs (**c**) lobular IBCs and SDBCs. The colour scale corresponds to the total fraction of detected mutations that are clonal, with a fraction of 1 indicating that every detected mutation is clonal and a fraction of 0 indicating that every detected mutation is subclonal. The clonal fraction is defined as N_clonal_/(N_clonal_+N_subclonal_), where N is the number of detected mutations in the gene across the cohort. ** significant difference in mutational frequency imposing a Bonferroni adjusted *P*-value of 7.46 ×10^−4^, * a significant difference at an unadjusted *P-*value of 0.05. § a significant difference in clonal fraction at an unadjusted *P*-value of 0.05.

In the 67 putative invasive breast cancer drivers identified in the 100kGP data, we identified 539 predicted oncogenic mutations in IBCs and 931 predicted oncogenic mutations in SDBCs^11^. As expected from previously published studies of breast cancer in both IBCs and SDBCs tumours^12^ the most commonly mutated breast cancer driver genes included *PIK3CA, TP53, KMT2C, GATA3, CDH1, MAP3K1* and *PTEN* (Supplementary Data 3). Furthermore, mutational frequencies in drivers differed significantly between ductal and lobular breast cancer histologies irrespective of screen detection status (Supplementary Fig. 2). There were, however, significant differences in the frequency of mutations in these driver genes between all IBCs and all SDBCs and when the analysis was only confined to ductal tumours (Fig. 3). For completeness we examined for differences in the frequency of mutations in all genes between IBC and SDBC, finding no further significant differences (Supplementary Data 4).

The frequency of *TP53* mutations was higher in IBCs compared with SDBCs (36% vs 24%, OR = 1.80, 95% CI: 1.29–2.51, *P* = 3.64 × 10^−4^), while the frequency of PIK3CA (33% vs 45%, OR: 0.59, 95% CI: 0.43–0.80, *P* = 6.11 × 10^−4^) and *MAP3K1* mutations was lower (IBC 8% vs SDBC 17%, OR = 0.43, 95% CI: 0.25–0.71, *P* = 4.34 × 10^−4^) (Fig. 3). These differences in mutational frequency remained significant when the analysis was confined to ductal histology and non-TN breast cancers. No significant difference in the frequency of mutations in oncogenic driver genes was shown between lobular IBC and SDBC, likely a consequence of limited statistical power. When considering the clonality of driver mutations, only *TP53* mutations were significantly more likely to be clonal in IBCs (*P* = 0.04, OR = 3.43, 95% CI 1.02–14.96).

**Fig. 4:**
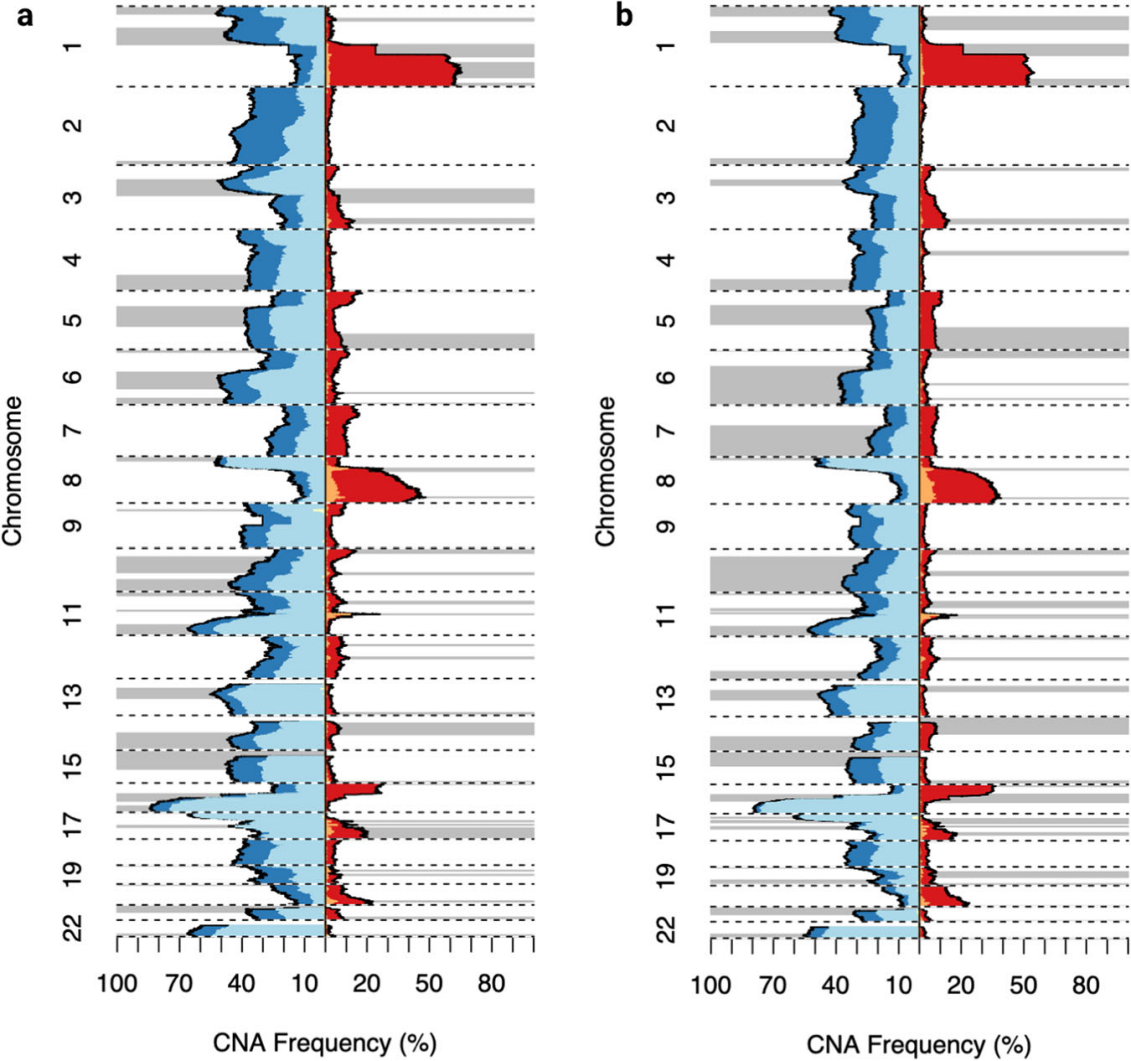
Copy number profile of IBC and SDBC. **a** IBC, **b** SDBC. Loss of heterozygosity is indicated in light blue, while other copy number losses are displayed in dark blue. Copy number gains are shown in red while large amplifications are indicated in orange. The regions with focal alterations, as defined by GISTIC2, are annotated with grey bars.

**Fig. 5:**
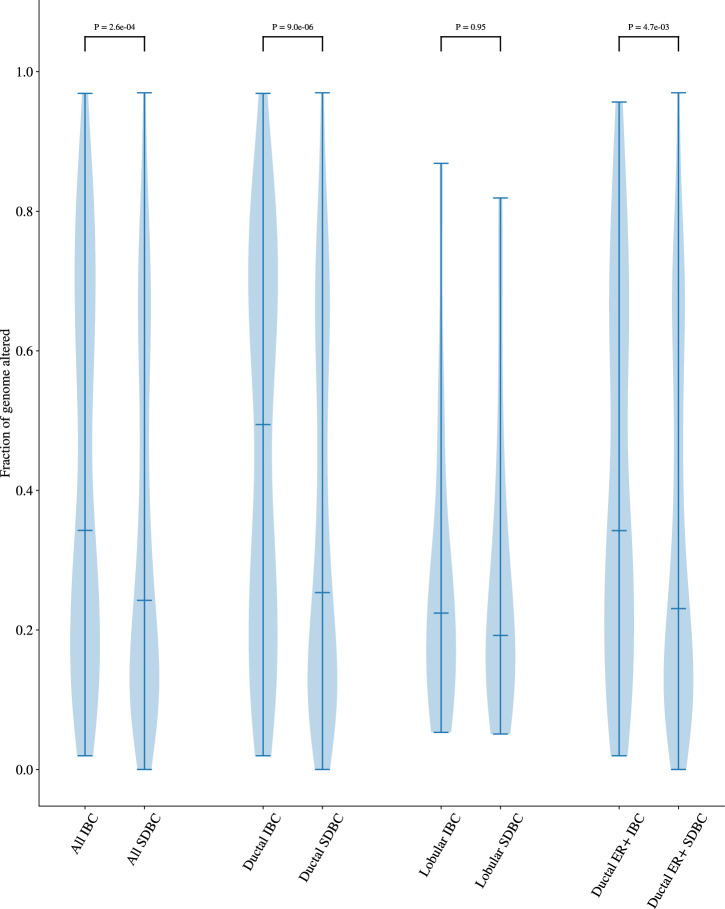
Proportion of the genome altered in IBC and SDBC. The distribution of the fraction of genome altered for IBCs and SDBCs. A region of the genome is considered to be altered if there is any deviation from a single copy of the major and minor allele. The median of the distribution is indicated by the horizontal line and *P*-values are calculated using a Wilcoxon rank sum test.

While the profile of somatic copy number alterations was broadly consistent between all IBCs and SDBCs (Fig. 4), the overall fraction of the genome altered was significantly higher in IBCs (*P* = 2.56 × 10^−4^). This difference remained significant when restricted to ductal (*P* = 9 × 10^−6^) and ER-positive (*P* = 4.67 × 10^−3^) subtypes (Fig. 5, Supplementary Fig. 3). Additionally, the number of CNAs was significantly higher in IBCs (*P* = 9.72 × 10^−4^) (Supplementary Data 5). Whole genome duplication (WGD) was also a significantly more frequent event for all (*P* = 8.51 × 10^−3^, OR = 1.59, 95% CI 1.11–2.27), ductal (*P* = 3.14 × 10^−4^, OR = 1.98, 95% CI 1.35–2.91) and non-TN IBCs (*P* = 0.01, OR = 1.76, 95% CI 1.11–2.79) compared to corresponding SDBCs. However, after adjusting for grade, the fraction of genome altered and number of CNAs were no longer significantly different between IBCs and SDBCs (*P* = 0.27, *P* = 0.50 respectively), reflecting the observation that IBCs are more likely to be higher grade.

Structural variants were a feature of almost all cancers (99% IBC vs 98% SDBC) and classification of SVs revealed that both balanced (*P* = 5.87 × 10^−3^, OR = 1.74, 95% CI 1.16–2.63) and unbalanced (*P* = 8.79 × 10^−3^, OR = 1.49, 95% CI 1.10–2.02) translocation events were enriched in IBCs. However, an adjustment for tumour grade greatly attenuated the effect such that only unbalanced translocations remained significantly enriched in IBCs (*P* = 0.03, OR = 1.03, 95% CI 1.00–1.06). No other patterns of structural rearrangements, including chromothripsis, were found to be statistically enriched or depleted in IBCs.

### Mutational signatures

To examine if the mutational processes in IBC and SDBC are different we utilised signature activities that have been previously extracted de novo and related to known COSMIC signatures^13^ (Supplementary Data 6, Supplementary Fig. 4). Across all cancers the most common signatures were those resulting from clock-like mutagenic processes (SBS1 and SBS5). As expected, signatures indicative of dHR were strongly associated with TN breast cancers, regardless of screening status. Across both IBC and SDBC the aetiological basis of dHR was only identifiable in 13% of cases on the basis of biallelic inactivation of *BRCA1, BRCA2, PALB2, BRIP1* or *RAD51B* through germline mutations, somatic mutations and loss of heterozygosity. A further 83% of cases harboured a monoallelic loss in these genes and hence many of the remaining cases may be caused by promoter methylation; however, this data is not available for 100kGP samples. Stratifying by screening status, dHR was significantly more common in IBC (OR = 2.55, 95% CI: 1.33–4.98, *P* = 3.38 × 10^−3^) and this association was primarily driven by ductal ER+ tumours (OR = 4.90, 95% CI: 1.79–14.81, *P* = 6.95 × 10^−4^). Moreover, signatures associated with dHR had significantly higher activities on average in IBC compared to SDBC: SBS3 (*P* = 0.02), ID6 (*P* = 1.79 × 10^−4^) and CN17 (*P* = 2.65 × 10^−3^). However, when restricting to TN breast cancers, dHR was not significantly enriched or depleted in IBCs (*P* = 0.66).

### Immune evasion

We predicted the presence of 12,785 neoantigens across both IBCs and SDBCs. There was no significant difference in neoantigen burden between IBCs and SDBCs (*P* = 0.26). The frequency of somatic mutations and LOH of HLA class I genes was not significantly different between IBCs and SDBCs. There was also no evidence to support preferential inactivation of an APG in IBC. Finally, there was no significant difference in the TCRA T-cell fraction between IBCs and SDBCs (*P* = 0.83).

## Discussion

In the 100kGP cohort, IBCs were characterised by more aggressive tumour characteristics than SDBCs - a higher histological grade, larger tumour size, more frequently lymph node-positive at diagnosis, a higher proportion of ER and PR negativity, and more likely to be TN. These observations are consistent with several published studies^7,14–31^. While not statistically significant, but in keeping with a recent study, IBC patients were more likely to be carriers of BRCA-mutations than those with SDBC (4.5% vs 2.5%)^32^.

Adjusting for grade, IBCs displayed a higher mutation rate while copy number abnormalities were not significantly different. IBCs additionally showed salient differences in driver mutational profile notably with a lower frequency of *PIK3CA* mutations and higher frequency of *TP53* mutations. These *TP53* mutations in IBCs were more likely to be clonal and hence by inference more likely to have arisen early in tumour development or confer a survival advantage to all tumour cells. This supports the notion that IBCs have differing evolutionary histories when compared to SDBCs^33,34^. The IBCs were also more likely to display dHR, which in part may reflect a higher rate of germline *BRCA* mutations. This is in keeping with published studies reporting a higher rate of germline HBOC predisposition in IBC compared to SDBC, as well as elevated rates of interval cancers in HBOC mutation carriers^9,32,35–40^. We did not, however, find evidence to indicate that women with IBC are at a lower PGS-defined breast cancer risk which has been reported by another analysis^41^. Similarly, while it has been proposed that IBC may be associated with a different immune response to SDBC^42^, our analysis of genetically mediated immune evasion or T-cell tumour infiltration provides no support for such an assertion.

Our findings are consistent with recently published studies reporting higher rates of *TP53* mutations and dHR in IBC^9,10^. Cheasley et al.^9^ also reported cancers in low mammographic density breasts (*n* = 142) had a higher frequency of *TP53* mutations, dHR, higher fraction of the genome altered, more copy number gains and were more likely to be interval breast cancers when compared to cancers in high mammographic density breasts (*n* = 119)^42^.

The major strengths of our study are having access to a unique dataset with high-coverage WGS data, linkage to high-quality clinicopathological information features on patients screened in the context of population-wide screening, thus avoiding biases related to screening indication. Furthermore, rather than focusing on a restricted set of genes we have been able to undertake a systematic analysis of the genetic landscape of IBCs. This has allowed us to assess tumour intrinsic mechanisms contributing to IBC as well as surrogates for the tumour microenvironment (TME).

We do, however, appreciate there are a number of limitations to our study. It is the case that while lobular cancers tend not to be a rapidly growing tumour, they are more likely to be missed by screening because of a diffuse growth pattern and minimal stromal response^43^. Indeed, in our analysis the mutational rate in lobular IBC was only marginal higher than in lobular SDBC. Hence, we acknowledge that a subset of lobular IBCs might not necessarily be true interval cancers but ones that are missed by screening. While some other cases in the IBC group may also be missed because of high breast density, it is noteworthy that the mutational rate in ductal IBCs was far higher than in ductal SDBC, which provides support for a different biology rather than solely being ascribable to the consequence of screening performance. Moreover in the absence of measured breast density we have relied on PGS in assessing the role of breast density and other modifiable factors, which inevitably affords limited power to demonstrate a relationship since these PGSs only capture a small proportion of the phenotypic variation of each risk factor^44,45^. Additionally, we did not have access to expression data and other classifiers of breast cancer histology such as the Gallen subtypes or PAM50^46,47^. Finally, we made use of indirect measurements of the TME and were unable to assess the impact of immune dysfunction in the earliest stages of tumour development.

As well as informing on the biology of IBCs, the findings of our study may inform on the detection and management of breast cancer. The higher number of *TP53* and copy number alterations as well as an increased mutation rate and dHR, suggests genomic instability has a greater role in IBCs when compared to SDBC. Genomic instability may be exploited therapeutically through synthetic lethality^48,49^, such as sensitivity to PARP in the context of *BRCA*-deficiency^50,51^. Furthermore, given 30% of breast cancers in women regularly attending screening are IBCs, additional screening modalities have been advocated as a means to advance the early detection of breast cancer. Whilst early in development, noninvasive detection of chromosomal instability in plasma circulating cell-free DNA has shown promise and may serve as an adjunct to breast screening mammography^52,53^.

Finally, our analysis highlights issues in the design of future studies investigating the biological basis of IBC. Specifically, there is a strong rationale for stratification of breast cancer cases based on measured breast density, specifically allowing for comparing subgroups to address confounding from mammographic density (i.e. study of ductal in low density breast, ductal in high density breasts, lobular in low density breast, lobular in high density breasts, each in IBC vs SDBC).

In conclusion, accepting the above caveats our findings indicate that while IBCs may not represent a distinct molecular subtype of breast cancer they are characterised by a more aggressive phenotype, in part likely to be a consequence of the timing of tumour initiation. Given that systematic review of national breast screening programs has found that IBCs are linked to worse patient survival^54^, as well as being pertinent to patient management, our findings have relevance to informing screening programs with respect to defining screening intervals.

## Methods

### Patients

This study was conducted as part of the 100 kGP which was granted ethical approval by the East of England – Cambridge South Research Ethics Committee. All patients provided written informed consent and the study was conducted in accordance with the Declaration of Helsinki. The 100 kGP project (release v14) provides WGS data on tumour-normal pairs from breast cancer patients recruited through 13 Genomic Medicine Centres across England. We restricted our WGS analysis to samples with high-quality data from PCR-free, flash-frozen fresh tumour samples (Supplementary Methods). Demographic and clinical data were obtained from Public Health England’s National Cancer Registration and Analysis Service (PHE-NCRAS), NHS Digital and the Genomic Medicine Centres. Tumour pathology information was obtained from histology reports. After QC (Supplementary Data 7), 833 of the 1488 patients with a diagnosis of breast cancer had a documented NHS screening history. To minimise bias associated with screening indication, we confined our analysis to breast cancers diagnosed through the NHS breast screening programme of women aged between 47 and 70 years (Table 1). Considering hormone receptor status, we confined our analysis to ductal histology since no patients with triple negative (TN) tumours had lobular breast cancer.

### Calling of somatic variants

In addition to using variant calls from the 100kGP analysis pipeline we: (i) removed alignment bias introduced by ISAAC soft clipping of semi-aligned reads^55^; (ii) called tumour copy number using Battenberg^33^; (iii) called structural variants (SVs) from a consensus of Manta^56^, LUMPY^57^, and DELLY^58^; (iv) removed insertions and deletions within 10 bp of a common germline indel (Supplementary Methods)^59^.

### Driver gene identification

To compare the frequency and clonality of driver gene mutations between IBCs and SDBCs we considered all driver genes identified across the 100 kGP cohort of 1488 invasive primary breast cancers. We identified cancer driver genes in these 1488 cases using IntOGen^60^, which combines seven computational methods to detect signals of positive mutational selection of missense mutations in coding regions of the genome (Supplementary Methods). Details of pre-processing of mutations, combining driver gene identification methodologies, post-processing and annotation of driver gene mutations are provided in Supplementary Methods.

### Signature analysis

de novo extraction of single-base-substitution (SBS), doublet-base-substitution (DBS), insertion and deletion (ID) signatures, copy number (CN) signatures and structural variant (SV) signatures including decomposition to known COSMIC signatures^13^ (v3.3), was performed using SigProfilerExtractor^61^. We used the extraction performed by Everall et al.^62^ and complemented this analysis by screening for evidence of homologous recombination deficiency (dHR) in tumours using HRDetect^63^; using the advocated probability threshold of 0.7 to classify tumours as exhibiting dHR.

### Estimation of clonality

The clonal state of driver mutations were estimated by MutationTimeR^34^. Sample odds ratios (ORs) were calculated for both early/late and clonal/subclonal driver mutations with associated *P*-values calculated using Fisher’s exact test. The ratio of subclonal to clonal mutations was used as a proxy for intratumor heterogeneity.

### Immune evasion

Neoantigens were identified using pVAC-Seq^64^ by predicting the binding affinities of epitopes that arise as a result of non-synonymous mutations, based on the HLA-alleles typed by POLYSOLVER^65^. We investigated three possible immune escape mechanisms, specifically: (i) non-synonymous mutation in any of the three (HLA-A,-B,-C) HLA Class I genes; (ii) loss of heterozygosity (LOH) in any of the three HLA-I genes or (iii) any inactivating mutation in one of the 22 antigen-presenting genes (APGs) involved in the IFN-γ pathway, the PF-L1 receptor, the CD58 receptor, and epigenetic escape via *SETDB1* (*APLNR, B2M, CANX, CALR, CD274, CD58, CIITA, ERAP1, ERAP2, IRF2, IFNGR1, IFNGR2, JAK1, JAK2, NLRC5, PDIA3, RFX5, SETDB1, STAT1, TAPBP, TAP1, TAP2*)^66,67^. We considered a tumour sample with positive immune escape status if it exhibited any one of (i)-(iii). HLA gene mutations were found using POLYSOLVER and LOH at HLA was predicted using LOHHLA^68^. We estimated numbers of tumour infiltrating lymphocytes based on the somatic copy number in conjunction with read depth of the T-cell receptor-α gene^69^.

### Germline variants and polygenic scores

We examined the germline of the patients for pathogenic variants in any of the 14 well-established hereditary breast ovarian cancer (HBOC) susceptibility genes (*ATM*, *BARD1*, *BRCA1*, *BRCA2*, *BRIP1*, *CDH1*, *CHEK2*, *MSH6*, *PALB2*, *PTEN*, *RAD51C*, *RAD51D*, *STK11* and *TP53*). Assignment of pathogenicity was based on a CADD^70^ score >30 and ClinVar annotation^71^. Genomic positions of canonical gene transcripts were retrieved from the Ensembl database (EnsDb.Hsapiens.v86)^72^ and were referenced to build GRCh38.

To generate polygenic scores (PGS) (Supplementary Methods) we used genome-wide association studies (GWAS) summary statistics estimated in European populations for breast cancer risk reported by Mavaddat et al.^73^. For the well-established modifiable risk factors for breast cancer, we used results from the following resources: GSCAN consortium meta-analysis of smoking initiation (ever vs never status)^74^ UK biobank (UKBB) meta-analysis of body mass index (BMI)^44^, summary statistics relating to breast density reported by Chen et al.^75^, and those relating to diabetes, such as fasting glucose and fasting insulin, were obtained from UKBB studies^76^.

### Statistical analysis

The relationship between categorical variables was assessed using either Chi-square or Fisher exact tests. Linear and logistic regression were used in analysis of continuous traits. The Kolmogorov–Smirnov test was performed to compare cumulative differences in PGS profiles. All statistical analysis we performed using R Version 4.2 and we considered a two-sided *P*-value < 0.05 as statistically significant.

### Reporting summary

Further information on research design is available in the Nature Research Reporting Summary linked to this article.

### Supplementary information

Supplementary Information

Supplementary Data 1-7

Reporting summary

## Data Availability

The data supporting the findings of this study are available within the Genomics England Research Environment, a secure cloud workspace. An example for details on how to access data for this publication can be found at https://re-docs.genomicsengland.co.uk/pan_cancer_pub/. Additional processed aggregated data supporting the findings presented in this manuscript can be found in the Supplementary Data. To access genomic and clinical data within this Research Environment, researchers must first apply to become a member of either the Genomics England Research Network (https://www.genomicsengland.co.uk/research/academic) or the Discovery Forum (industry partners https://www.genomicsengland.co.uk/research/research-environment). The process for joining the network is described at https://www.genomicsengland.co.uk/research/academic/join-gecip and consists of the following steps: 1. Your institution will need to sign a participation agreement available at https://files.genomicsengland.co.uk/documents/Genomics-England-GeCIP-Participation-Agreement-v2.0.pdf and email the signed version to gecip-help@genomicsengland.co.uk. 2. Once you have confirmed your institution is registered and have found a domain of interest, you can apply through the online form at https://www.genomicsengland.co.uk/research/academic/join-gecip. Once your Research Portal account is created you will be able to login and track your application. 3. Your application will be reviewed within 10 working days. 4. Your institution will validate your affiliation. 5. You will complete online Information Governance training and will be granted access to the Research Environment within 2 days of passing the online training. Data that has been made available to registered users include: alignments in BAM or CRAM format, annotated variant calls in VCF format, signatures assignment, tumour mutation burden, sequencing quality metrics, summary of findings that is shared with Genomic Lab Hubs, secondary clinical data as described in this paper. Further details of the types of data available (for example, mortality, hospital episode statistics and treatment data) can be found at https://re-docs.genomicsengland.co.uk/data_overview/. Germline variants can be explored in Interactive Variant Analysis Browser (see description at https://re-docs.genomicsengland.co.uk/iva_variant/). Cancer patients cohort and longitudinal clinical information on treatment and mortality can be explored with Participant Explorer (see description at https://re-docs.genomicsengland.co.uk/pxa/).

## Code availability

Code used in the generation of results and figures reported in this study can be found within the Genomics England Research Environment in the following directory: /re_gecip/shared_allGeCIPs/cmills/publications/breast_interval.
