# Supplementary material for: Genetic landscape of interval and screen detected breast cancer
Source: NPJ Precis Oncol. 2024 May 28;8:122. doi: 10.1038/s41698-024-00618-6 (PMC11133314; doi:10.1038/s41698-024-00618-6)
Supplement: Supplementary file 1 — Supplementary Information [file 41698_2024_618_MOESM1_ESM.pdf]

# Supplementary methods


**1. Sample curation** **2**
**2. Whole genome sequencing** **2**
**3. Small variant calling** **2**
**4. Copy number alterations** **3**
**5. Structural variant calling** **6**
**6. Whole genome duplication** **6**
**7. Identification of driver mutations** **7**
**8. Assessment of immune evasion** **8**

## 1. Sample curation

Tumour and germline sequencing data were obtained using version 14 of the main programme release of the 100,000 Genomes Project (100kGP), an NHS initiative for high-throughput sequencing of cancers and rare diseases[1,2]. Participant recruitment was coordinated by 13 Genomic Medicine Centres (GMCs) and affiliated hospitals from around the UK and written informed consent was provided by all patients. Ethical approval for the 100kGP was granted to Genomics England by the East of England – Cambridge South Research Ethics Committee (REC reference: 14/EE/1112). Tissue collection and the subsequent preparation, extraction and quantification of DNA was undertaken locally. Cubes or slices of tissue were cut from the tumour with a requirement that at least 40% of the tissue nuclei were malignant, and less than 20% of the area being necrotic. A minimum of 2μg of tumour DNA and 10μg of germline DNA was required for processing. DNA was then transferred to a central biorepository where whole genome sequencing of the paired tumour/normal DNA was performed by Illumina. The resulting processed BAM files were delivered to Genomics England, who performed additional quality checks.

## 2. Whole genome sequencing

Samples were prepared using the Illumina TruSeq DNA PCR-free library preparation kit and sequencing was performed using HiSeq X, producing 150 base pair (bp) paired-end reads. The average sequencing depth of normal blood and tumour samples were 30x and 100x respectively. Samples identified as having poor sequencing quality, considering: AT/CG dropout; percentage of mapped reads; percentage of chimeric DNA fragments; average insert size; local coverage unevenness, were excluded. Alignment of sequences to the *Homo sapiens* GRCh38Decoy assembly was achieved using Isaac (version 03.16.02.19)[3].

## 3. Small variant calling

Germline SNVs and indels were called using Starling[3] while somatic SNVs and small indels were called using Strelka (version 2.4.7)[4]. Somatic variants were removed using the default Strelka filters along with the following additional criteria:

- A population germline allele frequency ≥1% according to gnomAD[5] or the 100kGP dataset.
- A somatic frequency ≥5% in the 100kGP cancer dataset.
- Classified as a simple repeat by Tandem Repeats Finder[6].
- An indel for which ≥10% of base calls in a window of 50 bases on either side of the indel have been filtered by Strelka. This filter is indicative of a high level of sequencing noise.
- A majority of overlapping 150bp reads that align to multiple loci will result in those reads being discarded.
- Statistical evidence of variant being a result of a calling artefact. This was evaluated by comparing the ratio of tumour allele depths at a given site to that of allele depths in a panel of normal samples present in the 100kGP dataset. Individuals that did not carry the relevant alternate allele at a given site were included when computing the normal allele depth. In order to replicate the Strelka default filters, duplicated reads were removed and quality thresholds were set at base quality ≥5 and mapping quality ≥5. Furthermore, variants with a phred score <80, as calculated using Fisher's exact test, were removed.

High impact pathogenic germline variants were identified by further filtering based on CADD[7] scores and ClinVar[8] annotations. A threshold of CADD score > 30 was imposed, while variants were required to have a ClinVar annotation that was not "Benign".

## 4. Copy number alterations

Copy number alterations (CNAs) were called using an iterative procedure that utilised Battenberg v2.2.8[9]. The four steps to call CNAs are as follows:

**Step 1**) *Clonal and subclonal CNAs were profiled using Battenberg, along with an estimation of sample purity and tumour ploidy*. Briefly, the number of reads supporting SNV reference and alternate alleles were counted for both tumour and normal samples, using alleleCount-FixVAF[10]. Heterozygous SNVs

were phased using SHAPEIT2 v2.r904[11]. The phased SNVs were then segmented using piece-wise constant fitting[12] and subclonal segments were identified using t-tests. Sample purity and tumour ploidy were estimated using the method described by Van Loo, *et al.*[13] As sequencing data were aligned to hg38, it was necessary to convert SNV positions to hg37 before phasing, and convert output segments back to hg38.

**Step 2**) *Variant allele frequency distributions are used to evaluate copy number profile concordance*. The expected variant allele frequency is dependent on a number of factors, including: the fraction of tumour cells containing the variant; the tumour copy number profile; the number of chromosome copies with the variant (multiplicity); the sample purity[14]. Given the tumour copy number profile and an estimated sample purity, both estimated by Battenberg in step 1, we can expect to observe enrichment of variants with allele frequencies approximating particular values which represent clonal variants present in all tumour cells[13]. A failure to observe such an enrichment would suggest that either the copy number profile or sample purity is incorrect. We therefore assessed the Battenberg output validity via a comparison with the SNV variant allele frequency (VAF) distributions.

We considered only autosomal genome segments with copy number states of 1:1, 1:0, 2:2, 2:1, and 2:0, with no evidence of subclonal copy number states, when evaluating the SNV VAF distributions. Each of these five copy number states was evaluated separately, as the possible variant multiplicities and expected clonal SNV VAFs differ between states[13]. Copy number states which corresponded to genomic regions containing <5% of all SNVs were not considered. Expected locations of peaks in the VAF distribution were estimated as:

$$\frac{\rho_{Battenberg}m}{2(1-\rho_{Battenberg})+\rho_{Battenberg}\psi_v}$$

where $\rho_{Battenberg}$ is the sample purity as estimated by Battenberg, $\psi_v$ is the ploidy of the tumour at the variant site, and $m$ is the variant multiplicity. The multiplicity can take the value of 1 or 2 in copy number states of 2:2, 2:1 and 2:0, and only 1 in the remaining states. VAF distribution peaks were called using peakPick v0.11[15], which utilises kernel density estimation. Peaks that corresponded to a density <0.3 were excluded. Iterating over the copy number states, the expected location of the peak corresponding to the highest variant multiplicity was matched to the largest VAF of the observed distribution. All other expected peaks were matched to the observed peak with the most similar VAF. Tumour heterogeneity must be considered as it can reduce VAF peak detection capabilities.

Therefore, for samples where ≥1 expected peak locations were considered, the expected peak that was furthest from the respective matched observed peak was removed. Sample purity, $\rho_i$, was re-estimated for each remaining expected peak location (with VAF $a$) using the matched observed peak VAF, $\omega$:

$$\rho_i = \frac{2a}{m + \omega(2 - \psi_s)}$$

where $\psi_s$ is the ploidy of the respective copy number state. This allowed a single new purity estimate, $\rho_{new}$, to be computed as the weighted average of the peak-wise purity estimates:

$$\rho_{new} = \sum_i \frac{n_i \rho_i}{N q_i}$$

where $n_i$ is the number of SNVs in genomic regions with the copy number state, *N* is the number of SNVs in genomic regions of all considered copy number states, and $q_i$ is the number of considered multiplicities for the copy number state.

**Step 3**) *Quality assessment of CNA profiles.* A metric to aid in evaluating the CNA profile quality is the weighted average of the difference between the purity estimated by Battenberg and the peak-wise purity estimates:

$$\eta = \sum_i \frac{n_i |\rho_i - \rho_{Battenberg}|}{N q_i}.$$

The following criteria were used to evaluate the CNA profiles:

- The location of VAF distribution peaks were estimated correctly (defined as $\eta$ <5%).
- DPClust[9] identified a cluster containing ≥5% of all SNVs with a CCF of between 0.9 and 1.1.
- DPClust identified no clusters containing ≥5% of all SNVs with CCFs >1.1.
- In the case that Battenberg estimates that most of the genome is tetraploid (2:2), a peak in the SNV VAF distribution in 2:2 regions corresponding to a variant multiplicity of 1 was observed.
- No single homozygous deletion >10Mb is called.

Samples satisfying all criteria were deemed to pass and their CNA profiles and purity estimates were used in downstream analyses. Samples that did not pass these criteria were re-profiled (**Step 4**).

**Step 4**) CNA re-profiling occurred a maximum of three times using Battenberg with new purity and ploidy estimates. Samples that did not pass the quality assessment criteria (**Step 3**) after three re-

profiling attempts were not considered in downstream analyses. The new purity, $\rho_{new}$, was estimated in **Step 2**, whilst the new ploidy, $\psi_{new}$, was estimated using[13]:

$$\psi_{new} = \frac{\rho_{Battenberg}\left(\psi_{Battenberg} - 2\right) + 2\rho_{new}}{\rho_{new}}$$

## 5. Structural variant calling

Somatic structural variants (SVs) were called using Delly[16], Lumpy[17] and Manta[18], taking a consensus of all three while also taking into account the identified copy number alterations. The default parameters were used for all three SV callers. In addition, Delly was run with post-filtering of somatic SVs using all normal samples, as described in the Delly documentation. SVs from the three individual callers were subject to the following additional filters and removed if satisfying any condition: any reads supporting the variant were also identified in the matched normal sample; the variant was supported by <2% of tumour reads; a variant breakpoint was located in a telomeric or centromeric region; a variant breakpoint was located on a non-standard reference contig (*i.e.,* not chromosomes 1-22, X or Y). The identified SVs were combined using a modified version of PCAWG Merge SV, which utilises a graph-based approach to identify and merge SVs identified by multiple callers, allowing a 400bp window for the breakpoint positions[19]. SVs were retained if they were identified by at least two of the SVs callers. Additionally, a SV was also retained if identified by a single SV caller but with a breakpoint that lies within 3kb of a called CNA segment boundary.

## 6. Whole genome duplication

The average genome copy number state, $\psi_{ave}$, was used as a metric to determine the threshold used to classify whether a tumour had undergone whole genome duplication (WGD). $\psi_{ave}$ is defined as

$$\psi_{ave} = \frac{\sum_{i=1}^{S}(L_i \sum_{j=1}^{2}(F_{i,j}(C_{j,i}^{Maj} + C_{j,i}^{Min})))}{\sum_{i=1}^{S} L_i},$$

where $S$ is the number of copy number genome segments, $F_{j,i}$ is the fraction of tumour cells carrying copy number state $j$, $C_{j,i}^{Maj}$ and $C_{j,i}^{Min}$ are the major and minor allele copy numbers and $L_i$ is the base

pair length of a genome segment. In the absence of a subclonal alteration, $F_{1,i} = 1$ and $F_{2,i} = 0$. WGD status is determined by[20]

$$\begin{array}{ll} WGD, & if\ 2.9 - 2H < \psi_{ave} \\ Not\ WGD, & if\ 2.9 - 2H > \psi_{ave} \end{array}$$

where $H$ is the fraction of the genome affected by loss of heterozygosity.

## 7. Identification of driver mutations

Candidate protein-coding driver genes were identified using The Integrative OncoGenomics pipeline (IntOGen)[21]. Tumour samples were flagged for exclusion from driver gene identification if they were hypermutated (>10,000 mutations) or had an outlier mutation count when compared to the rest of the cohort. This threshold was defined as: upper quartile + 1.5 × interquartile range. Additionally, mutations that were identified in the Hartwig Consortium Panel of Normal samples were also excluded[22].

IntOGen incorporates seven driver gene identification methods: dNdSCV[23]; OncodriveFML[24]; OncodriveCLUSTL[25]; cBaSE[26]; MutPanning[27]; HotMaps3D[28]; smRegions[29]. The results of each of the driver identification methods were combined by first generating a "truth set" of driver genes using Tier 1 and 2 genes in the COSMIC Cancer Gene Census[30]. The relative enrichment of genes in the truth set is used to generate a weighting for each method. A consensus ranking of driver genes was generated using Schulze's voting method, taking the ranked lists of genes from each of the seven methods. *P*-values were estimated using a weighted Stouffer Z-score method. With the consensus ranking and combined *P*-values estimated, driver genes were classified into four tiers of descending confidence: Tier 1 - genes where the consensus ranking is higher than the ranking of the first gene with Stouffer Q > 0.05; Tier 2 - genes which are part of the truth set and show a combined Stouffer Q < 0.25; Tier 3 - genes with a Stouffer Q < 0.05; Tier 4 - genes with Stouffer Q > 0.05. Candidate genes are classified according to their highest possible tiering, e.g a gene that satisfies the criteria for tiers 1 and 3 will be classified as tier 1.

Additional filtering was performed on the candidate driver genes as a final step of the identification process. Genes were excluded from the final list of driver genes if they had any of the following

properties: classified as tier 4 by the combination method; only significant (Q < 0.1) in one of the seven identification methods; the gene has very low expression in The Cancer Genome Atlas breast cancer samples; the gene is in a list of olfactory receptor genes; the gene is in a known list of artefacts or long genes. Further details of the driver identification process are given by Kinnersley *et al*.[31]

## 8. Polygenic risk scores

Polygenic scores (PGS) were calculated on a per patient basis using genome-wide association studies (GWAS) summary statistics for European populations. Scores were calculated by plink using the '--scores' argument with the 'sum' argument enabled. The per patient PGS were aggregated and normalised such that the distribution for the whole cohort had mean of 0 and standard deviation of 1. The normalised scores for IBCs and SDBCs were extracted and their difference assessed using a t test.

We used the GWAS as reported by Mavaddat et al.[32] for breast cancer risk. We also considered a number of well-established modifiable risk factors for breast cancer and used GWAS from the following resources: GSCAN consortium meta-analysis of smoking initiation (ever vs never status)32 UK biobank (UKBB) meta-analysis of body mass index (BMI)33, and those relating to diabetes, such as fasting glucose and fasting insulin, were obtained from UKBB studies35. We used GWAS relating to breast density as reported by Chen et al.34.

## 9. Assessment of immune evasion

**Neoantigen prediction**

HLA-typing was performed using POLYmorphic loci reSOLVER[33] (POLYSOLVER), resulting in all six HLA class I alleles (from the HLA-A, HLA-B and HLA-C genes) identified for all samples. Neoantigens were predicted using personalized Variant Antigens by Cancer Sequencing (pVAC-Seq)[34]. pVAC-Seq uses the predicted binding affinities of peptides, arising due to non-synonymous mutations, to major histocompatibility complex class I molecules. This is achieved by combining the results of eight

methods (NetMHC[35], NetMHCpan[36], MHCflurry[37], SMM[38], NetMHCcons[39], SMMPMBEC[40], MHCnuggets[41], PickPocket[42]) based on the HLA-alleles typed by POLYSOLVER.

Peptides are classified as neoantigens if the peptide meets the following conditions:

- Has a binding affinity ≤500nM (mean of all 8 methods).
- Corresponds to a canonical transcript.
- Is novel with respect to the human proteome.

**Immune escape mechanisms**

We considered three mechanisms of genetic immune escape: a non-synonymous mutation in any of the three HLA Class I genes; LOH in any of the three HLA-I genes; any inactivating mutation in a list of 22 genes essential to antigen presentation. POLYSOLVER was used to identify somatic mutations in the HLA genes. This uses a combination of MuTect[43] to check for non-synonymous SNVs and Strelka for insertions and deletions in HLA-aligned reads. HLA LOH was predicted using Loss of Heterozygosity in Human Leukocyte Antigen[44] (LOHHLA). LOHHLA was run with the number of mismatch sites between any two allele pairs set to >10 and the minimum coverage filter at these sites set to 10. LOHHLA did not make predictions of LOH for genes of patients that did not meet either of these thresholds, thus homozygous alleles were neglected. To define LOH of HLA we used the same definition as Cornish *et al.*[45]

- Presence of allelic imbalance, with the difference in evidence of the two alleles fulfilling $P$ <0.01.
- The copy number of the lost allele was <0.5 with a confidence interval <0.7.
- The copy number of the kept allele was >0.7.
- The number of mismatched sites between alleles was >10.

A list of 22 antigen presenting genes was created by considering the genetic components of antigen presenting machinery. Specifically, the IFN-$\gamma$ pathway, the PF-L1 receptor, the CD58 receptor, and epigenetic escape via *SETDB1* were considered resulting in the following genes[46,47]: *APLNR, B2M, CANX, CALR, CD274, CD58, CIITA, ERAP1, ERAP2, IRF2, IFNGR1, IFNGR2, JAK1, JAK2, NLRC5, PDIA3, RFX5, SETDB1, STAT1, TAPBP, TAP1, TAP2*. A gene was classified as inactivated if any one of the following conditions was met:

- Monoalleic or bialleic somatic mutation annotated with any of the following VEP[48] consequences: 'frameshift variant', 'stop gained', 'stop lost', 'splice acceptor variant', 'splice donor variant', 'splice region variant' or 'start lost'.
- Biallelic somatic mutation, or a monoalleic somatic mutation plus LOH, annotated with any of the following VEP consequences: 'transcript ablation', 'transcript amplification', 'inframe insertion', 'inframe deletion', 'missense variant' or 'protein altering variant'.
- Homozygous deletion.

## 10. Extraction of mutational signatures

*De novo* extraction of single-base-substitution (SBS), doublet-base-substitution (DBS), insertion and deletion (ID), copy number (CN) and structural variant (SV) signatures, including decomposition to known COSMIC signatures[49] (v3.3), was performed using SigProfilerExtractor[50] by Everall *et al.*[51] Single base substitutions were classified by considering their tri-nucleotide and transcriptional context, resulting in 288 unique classes. Double base substitutions and small indels were classified into 78 and 83 classes as is the case in COSMIC. Copy number events were classified into 48 classes that depended on the length of the sequence, type of copy number change and whether LOH was present[52]. Thirty two classes of SVs were based on the size of the SV and whether it existed as part of a cluster[53].

All signatures were extracted using random initialization, 500 NMF replicates, and between 10,000 and 1,000,000 NMF iterations. We assumed the presence of between 1 and 25 SBS and ID signatures, between 1 and 20 DBS signatures, and between 1 and 15 CN and SV signatures. Optimal solutions were manually chosen considering solution stability across NMF replicates and the observed mutational profile reconstruction error.

## References


1. Turnbull, C. Introducing whole-genome sequencing into routine cancer care: the Genomics England 100 000 Genomes Project. *Ann. Oncol.* **29**, 784–787 (2018).
2. Turnbull, C. *et al.* The 100 000 Genomes Project: bringing whole genome sequencing to the NHS. *BMJ* **361**, k1687 (2018).
3. Raczy, C. *et al.* Isaac: ultra-fast whole-genome secondary analysis on Illumina sequencing platforms. *Bioinformatics* **29**, 2041–2043 (2013).
4. Saunders, C. T. *et al.* Strelka: accurate somatic small-variant calling from sequenced tumor-normal sample pairs. *Bioinformatics* **28**, 1811–1817 (2012).
5. Karczewski, K. J. *et al.* The mutational constraint spectrum quantified from variation in 141,456 humans. *Nature* **581**, 434–443 (2020).
6. Benson, G. Tandem repeats finder: a program to analyze DNA sequences. *Nucleic Acids Res.* **27**, 573–580 (1999).
7. Rentzsch, P., Witten, D., Cooper, G. M., Shendure, J. & Kircher, M. CADD: predicting the deleteriousness of variants throughout the human genome. *Nucleic Acids Res.* **47**, D886–D894 (2018).
8. Landrum, M. J. *et al.* ClinVar: public archive of relationships among sequence variation and human phenotype. *Nucleic Acids Res.* **42**, D980–5 (2014).
9. Nik-Zainal, S. *et al.* The life history of 21 breast cancers. *Cell* **149**, 994–1007 (2012).
10. Cornish, A. J. *et al.* Reference bias in the Illumina Isaac aligner. *Bioinformatics* vol. 36 4671–4672 (2020).
11. Delaneau, O., Marchini, J. & Zagury, J.-F. A linear complexity phasing method for thousands of genomes. *Nat. Methods* **9**, 179–181 (2011).
12. Nilsen, G. *et al.* Copynumber: Efficient algorithms for single- and multi-track copy number segmentation. *BMC Genomics* **13**, 591 (2012).
13. Van Loo, P. *et al.* Allele-specific copy number analysis of tumors. *Proc. Natl. Acad. Sci. U. S. A.* **107**, 16910–16915 (2010).

14. Dentro, S. C., Wedge, D. C. & Van Loo, P. Principles of Reconstructing the Subclonal Architecture of Cancers. *Cold Spring Harb. Perspect. Med.* **7**, (2017).
15. Weber, C. M., Ramachandran, S. & Henikoff, S. Nucleosomes are context-specific, H2A.Z-modulated barriers to RNA polymerase. *Mol Cell* **53**, 819–830 (2014).
16. Rausch, T. *et al.* DELLY: structural variant discovery by integrated paired-end and split-read analysis. *Bioinformatics* **28**, i333–i339 (2012).
17. Layer, R. M., Chiang, C., Quinlan, A. R. & Hall, I. M. LUMPY: a probabilistic framework for structural variant discovery. *Genome Biol.* **15**, R84 (2014).
18. Chen, X. *et al.* Manta: rapid detection of structural variants and indels for germline and cancer sequencing applications. *Bioinformatics* **32**, 1220–1222 (2016).
19. Li, Y. *et al.* Patterns of somatic structural variation in human cancer genomes. *Nature* **578**, 112–121 (2020).
20. Gerstung, M. *et al.* The evolutionary history of 2,658 cancers. *Nature* **578**, 122–128 (2020).
21. Martínez-Jiménez, F. *et al.* A compendium of mutational cancer driver genes. *Nat. Rev. Cancer* **20**, 555–572 (2020).
22. Priestley, P. *et al.* Pan-cancer whole-genome analyses of metastatic solid tumours. *Nature* **575**, 210–216 (2019).
23. Martincorena, I. *et al.* Universal Patterns of Selection in Cancer and Somatic Tissues. *Cell* **173**, 1823 (2018).
24. Mularoni, L., Sabarinathan, R., Deu-Pons, J., Gonzalez-Perez, A. & López-Bigas, N. OncodriveFML: a general framework to identify coding and non-coding regions with cancer driver mutations. *Genome Biol.* **17**, 128 (2016).
25. Arnedo-Pac, C., Mularoni, L., Muiños, F., Gonzalez-Perez, A. & Lopez-Bigas, N. OncodriveCLUSTL: a sequence-based clustering method to identify cancer drivers. *Bioinformatics* **35**, 5396 (2019).
26. Weghorn, D. & Sunyaev, S. Bayesian inference of negative and positive selection in human cancers. *Nat. Genet.* **49**, 1785–1788 (2017).

27. Dietlein, F. *et al.* Identification of cancer driver genes based on nucleotide context. *Nat. Genet.* **52**, 208–218 (2020).

28. Tokheim, C. *et al.* Exome-Scale Discovery of Hotspot Mutation Regions in Human Cancer Using 3D Protein Structure. *Cancer Res.* **76**, 3719–3731 (2016).

29. Martínez-Jiménez, F., Muiños, F., López-Arribillaga, E., Lopez-Bigas, N. & Gonzalez-Perez, A. Systematic analysis of alterations in the ubiquitin proteolysis system reveals its contribution to driver mutations in cancer. *Nat Cancer* **1**, 122–135 (2020).

30. Sondka, Z. *et al.* The COSMIC Cancer Gene Census: describing genetic dysfunction across all human cancers. *Nat. Rev. Cancer* **18**, 696–705 (2018).

31. Kinnersley, B. *et al.* Cancer driver genes and opportunities for precision oncology revealed by whole genome sequencing 10,478 cancers. *medRxiv* 2023.05.24.23289454 (2023) doi:10.1101/2023.05.24.23289454.

32. Mavaddat, N. *et al.* Prediction of breast cancer risk based on profiling with common genetic variants. *J. Natl. Cancer Inst.* **107**, (2015).

33. Shukla, S. A. *et al.* Comprehensive analysis of cancer-associated somatic mutations in class I HLA genes. *Nat. Biotechnol.* **33**, 1152–1158 (2015).

34. Hundal, J. *et al.* pVAC-Seq: A genome-guided in silico approach to identifying tumor neoantigens. *Genome Med.* **8**, 11 (2016).

35. Andreatta, M. & Nielsen, M. Gapped sequence alignment using artificial neural networks: application to the MHC class I system. *Bioinformatics* **32**, 511–517 (2015).

36. Reynisson, B., Alvarez, B., Paul, S., Peters, B. & Nielsen, M. NetMHCpan-4.1 and NetMHCIIpan-4.0: improved predictions of MHC antigen presentation by concurrent motif deconvolution and integration of MS MHC eluted ligand data. *Nucleic Acids Res.* **48**, W449–W454 (2020).

37. MHCflurry: Open-Source Class I MHC Binding Affinity Prediction. *Cell Systems* **7**, 129–132.e4 (2018).

38. Peters, B. & Sette, A. Generating quantitative models describing the sequence specificity of biological processes with the stabilized matrix method. *BMC Bioinformatics*

**6**, 1–9 (2005).

39. Karosiene, E., Lundegaard, C., Lund, O. & Nielsen, M. NetMHCcons: a consensus method for the major histocompatibility complex class I predictions. *Immunogenetics* **64**, 177–186 (2011).
40. Kim, Y., Sidney, J., Pinilla, C., Sette, A. & Peters, B. Derivation of an amino acid similarity matrix for peptide:MHC binding and its application as a Bayesian prior. *BMC Bioinformatics* **10**, 1–11 (2009).
41. Shao, X. M. *et al.* High-Throughput Prediction of MHC Class I and II Neoantigens with MHCnuggets. *Cancer Immunol Res* **8**, 396–408 (2020).
42. Zhang, H., Lund, O. & Nielsen, M. The PickPocket method for predicting binding specificities for receptors based on receptor pocket similarities: application to MHC-peptide binding. *Bioinformatics* **25**, 1293–1299 (2009).
43. Cibulskis, K. *et al.* Sensitive detection of somatic point mutations in impure and heterogeneous cancer samples. *Nat. Biotechnol.* **31**, 213–219 (2013).
44. Allele-Specific HLA Loss and Immune Escape in Lung Cancer Evolution. *Cell* **171**, 1259–1271.e11 (2017).
45. Cornish, A. J. *et al.* Whole genome sequencing of 2,023 colorectal cancers reveals mutational landscapes, new driver genes and immune interactions. *bioRxiv* 2022.11.16.515599 (2022) doi:10.1101/2022.11.16.515599.
46. Kelly, A. & Trowsdale, J. Genetics of antigen processing and presentation. *Immunogenetics* **71**, 161–170 (2018).
47. Martínez-Jiménez, F. *et al.* Genetic immune escape landscape in primary and metastatic cancer. *Nat. Genet.* **55**, 820–831 (2023).
48. McLaren, W. *et al.* The Ensembl Variant Effect Predictor. *Genome Biol.* **17**, 1–14 (2016).
49. Tate, J. G. *et al.* COSMIC: the Catalogue Of Somatic Mutations In Cancer. *Nucleic Acids Res.* **47**, D941–D947 (2018).
50. Uncovering novel mutational signatures by de novo extraction with SigProfilerExtractor.

*Cell Genomics* **2**, 100179 (2022).

51. Everall, A. *et al.* Comprehensive repertoire of the chromosomal alteration and mutational signatures across 16 cancer types from 10,983 cancer patients. *medRxiv* 2023.06.07.23290970 (2023) doi:10.1101/2023.06.07.23290970.
52. Steele, C. D. *et al.* Signatures of copy number alterations in human cancer. *Nature* **606**, 984–991 (2022).
53. Degasperi, A. *et al.* A practical framework and online tool for mutational signature analyses show inter-tissue variation and driver dependencies. *Nat Cancer* **1**, 249–263 (2020).

# Supplementary figures

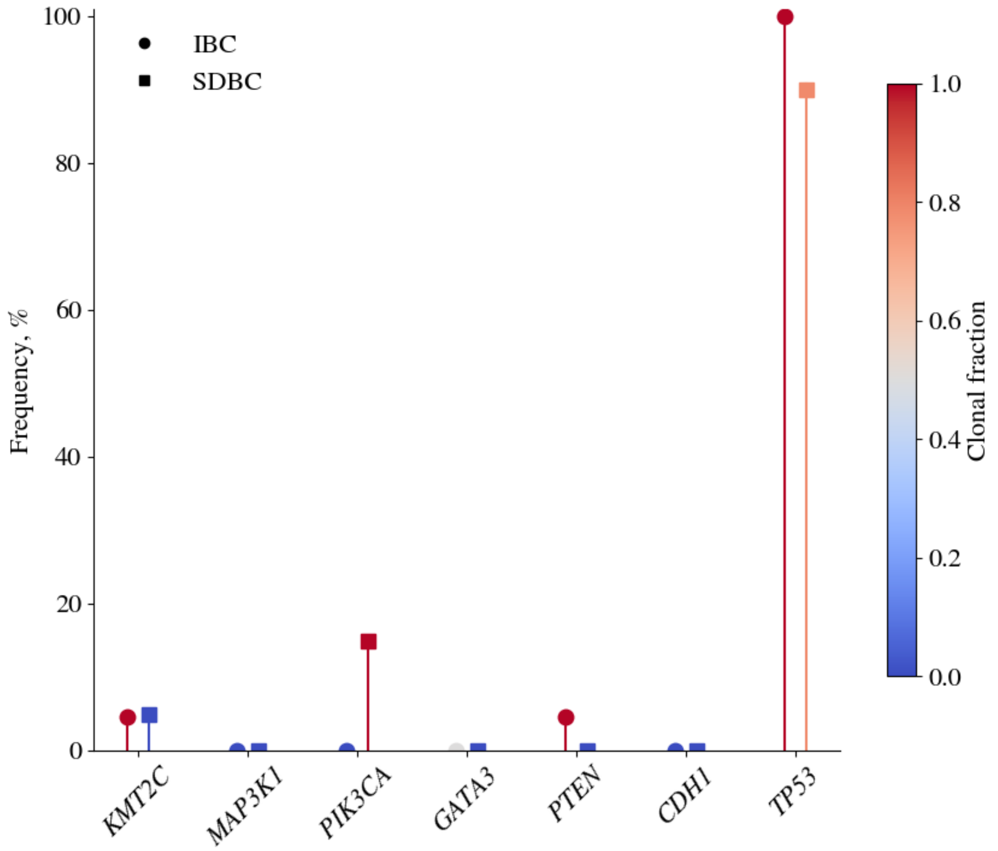


**Supplementary figure 1. Frequency and clonality of driver gene mutations in ductal triple negative breast cancers.** The mutational frequency of driver genes for triple negative ductal breast cancers. The colour scale corresponds to the total fraction of mutations that are clonal. Genes labelled with ** show a significant difference in frequency imposing a Bonferroni adjusted *P*-value of $7.46 \times 10^{-4}$, and those labelled with * a significant difference at an unadjusted *P*-value of 0.05.

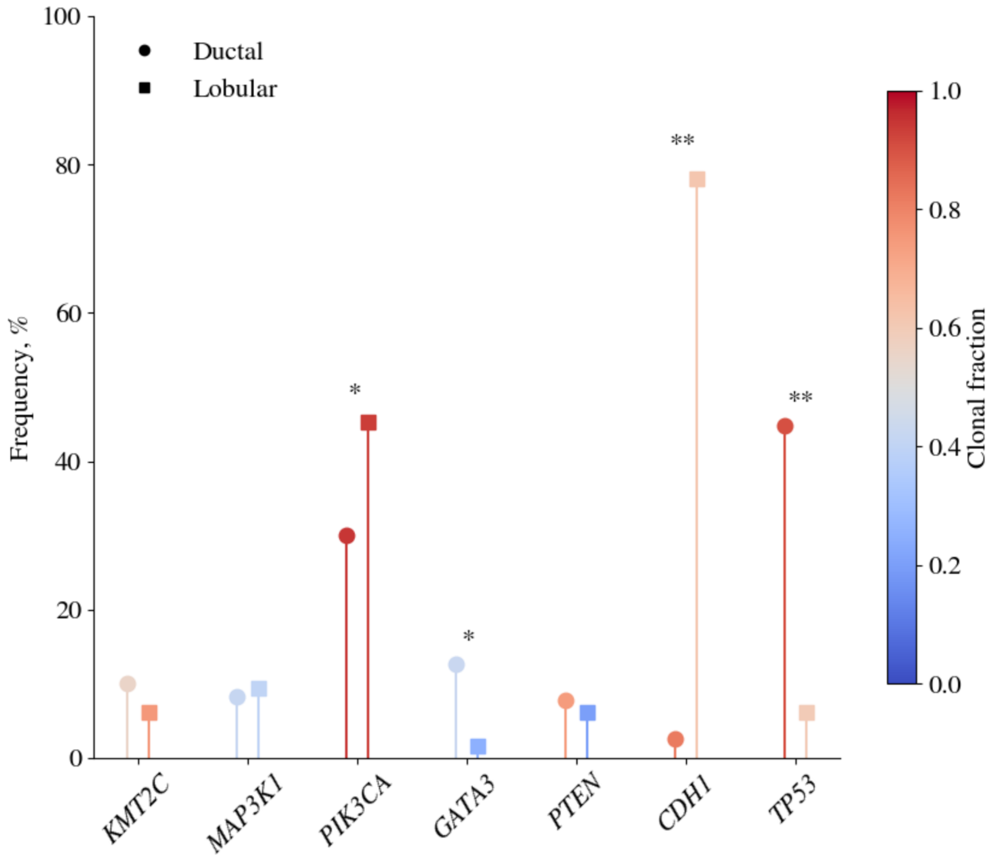


**Supplementary figure 2. Frequency and clonality of driver gene mutations in ductal breast cancers and lobular breast cancers.** The mutational frequency of driver genes for all ductal breast cancers and all lobular breast cancers, irrespective of screening status. The colour scale corresponds to the total fraction of mutations that are clonal. Genes labelled with ** show a significant difference in frequency imposing a Bonferroni adjusted *P*-value of $7.46 \times 10^{-4}$, and those labelled with * a significant difference at an unadjusted *P*-value of 0.05.

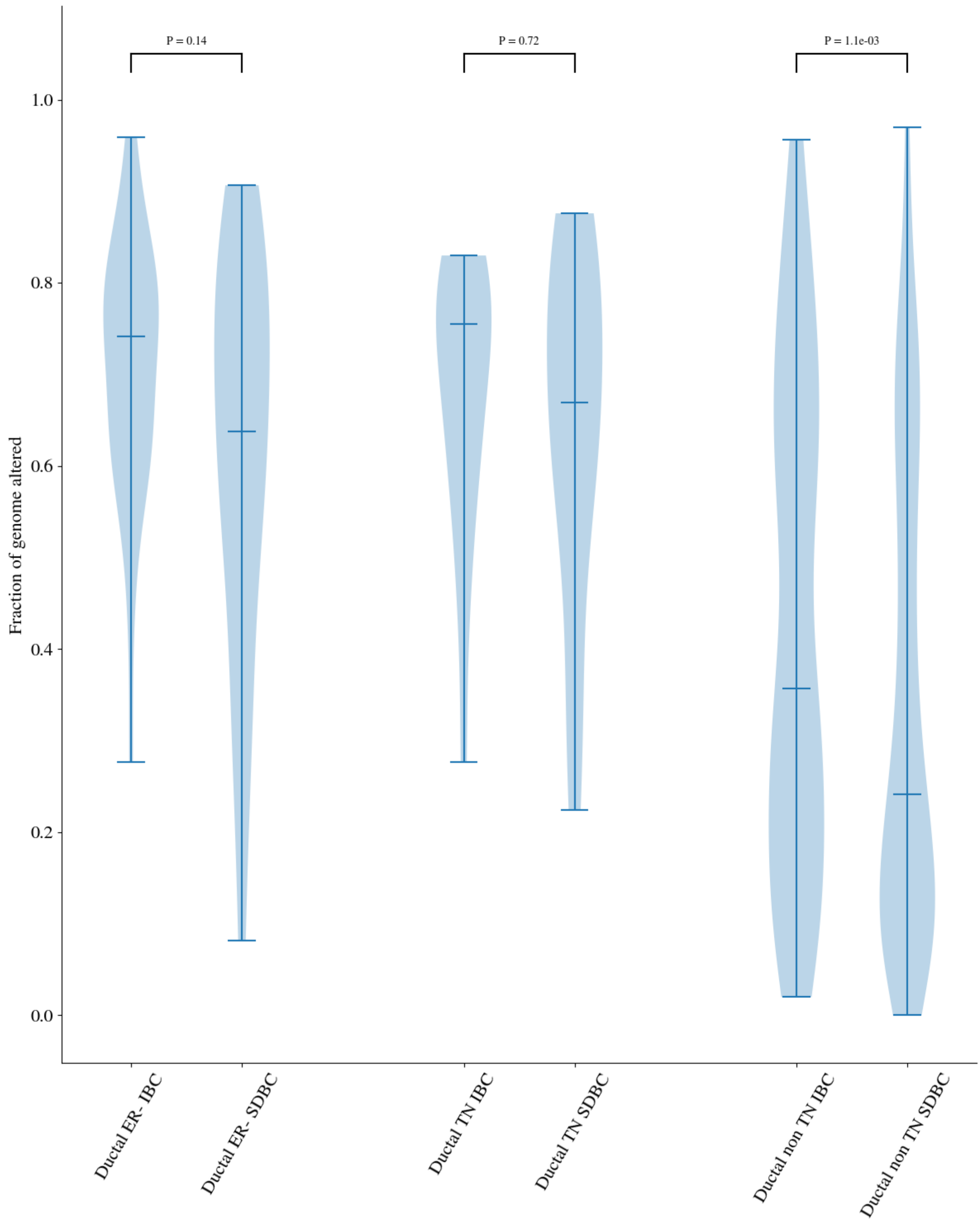


**Supplementary figure 3. Fraction of genome altered.** The fraction of genome altered for ductal ER-, ductal triple negative and ductal non triple negative tumours. The median of the distribution is indicated by the horizontal line and *P*-values calculated using a Wilcoxon rank sum test.

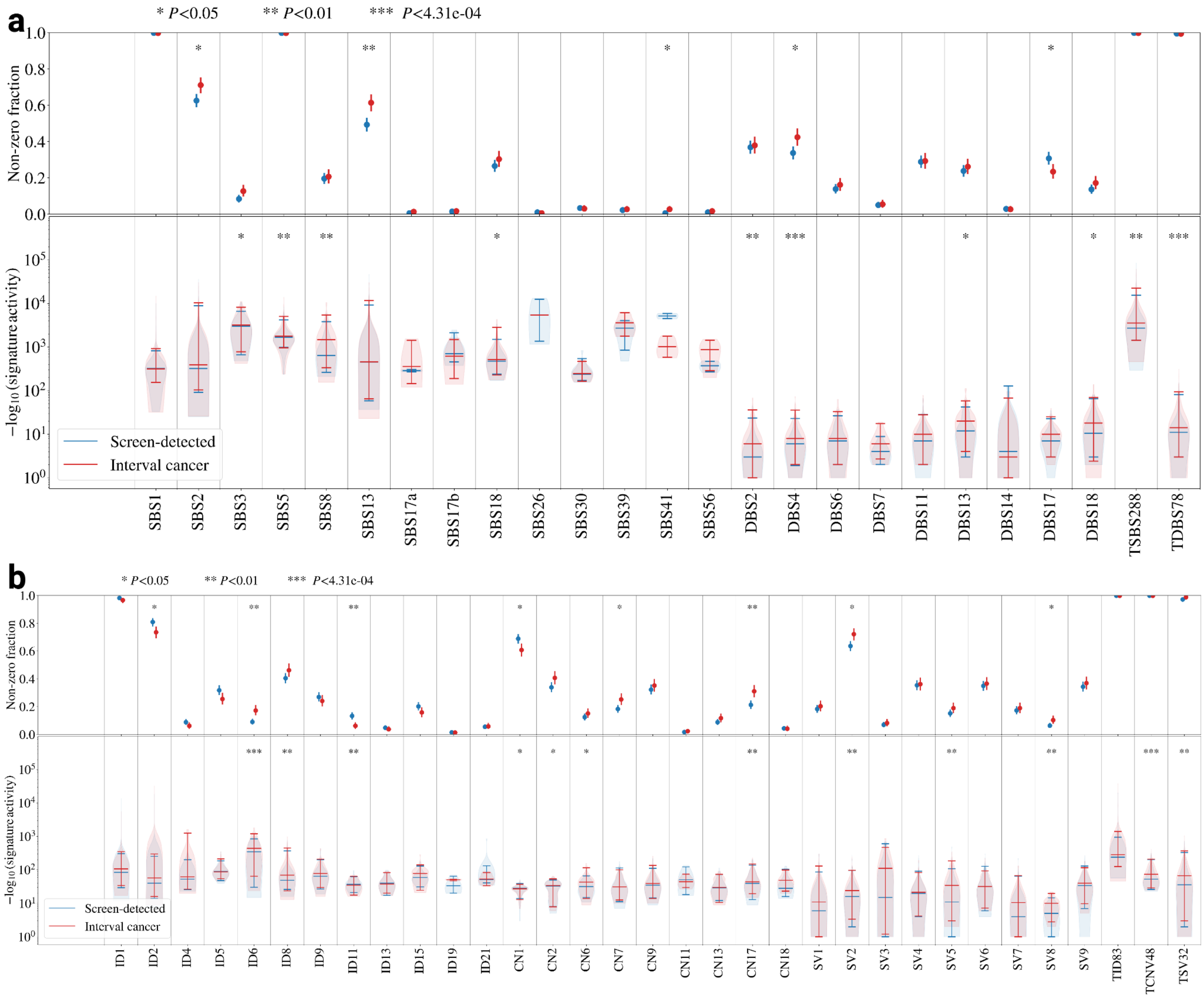


**Supplementary figure 4. Frequency and activity of mutational signatures. a**) Single base substitution (SBS) and doublet base substitution (DBS) signatures **b**) Insertion-deletion (ID), copy number (CN) and structural variant (SV) signatures. Each signature displayed has been extracted in at least one sample in this cohort. The proportion of samples with the given signature are displayed in the upper plot, while the distribution of signature activities are displayed in the lower plot. *P*-values are estimated using a binomial test for the fraction of samples displaying a given signature while a t-test is used to compare the signature activities. Signatures prefixed with "T" indicate the sum of all extracted signatures for the given substitution type.
