# Supplementary material for: Genetic landscape of interval and screen detected breast cancer
Source: NPJ Precis Oncol. 2024 May 28;8:122. doi: 10.1038/s41698-024-00618-6 (PMC11133314; doi:10.1038/s41698-024-00618-6)
Supplement: Supplementary file 3 — Reporting summary [file 41698_2024_618_MOESM3_ESM.pdf]

nature portfolio

Corresponding author(s): Richard Houlston

Last updated by author(s): May 2, 2024

Reporting Summary

Nature Portfolio wishes to improve the reproducibility of the work that we publish. This form provides structure for consistency and transparency in reporting. For further information on Nature Portfolio policies, see our Editorial Policies and the Editorial Policy Checklist.

Statistics

For all statistical analyses, confirm that the following items are present in the figure legend, table legend, main text, or Methods section.

n/a	Confirmed	
☐	☒	The exact sample size (*n*) for each experimental group/condition, given as a discrete number and unit of measurement
☐	☒	A statement on whether measurements were taken from distinct samples or whether the same sample was measured repeatedly
☐	☒	The statistical test(s) used AND whether they are one- or two-sided<br>*Only common tests should be described solely by name; describe more complex techniques in the Methods section.*
☐	☒	A description of all covariates tested
☐	☒	A description of any assumptions or corrections, such as tests of normality and adjustment for multiple comparisons
☐	☒	A full description of the statistical parameters including central tendency (e.g. means) or other basic estimates (e.g. regression coefficient) AND variation (e.g. standard deviation) or associated estimates of uncertainty (e.g. confidence intervals)
☐	☒	For null hypothesis testing, the test statistic (e.g. *F*, *t*, *r*) with confidence intervals, effect sizes, degrees of freedom and *P* value noted<br>*Give P values as exact values whenever suitable.*
☒	☐	For Bayesian analysis, information on the choice of priors and Markov chain Monte Carlo settings
☒	☐	For hierarchical and complex designs, identification of the appropriate level for tests and full reporting of outcomes
☒	☐	Estimates of effect sizes (e.g. Cohen's *d*, Pearson's *r*), indicating how they were calculated

Our web collection on statistics for biologists contains articles on many of the points above.

Software and code

Policy information about availability of computer code

Data collection	Data was collected and processed by Genomics England and Illumina, using the Issac aligner and using Starling and Strelka to call variants.
Data analysis	Mutations were annotated with VEP and ClinVar, and filtered based on CADD scores. Copy number alterations were called using Battenberg. Structural variants called using Lumpy, Manta and Delly. Driver mutations were identified using IntOGen. Polygenic scores were calculated using Plink, while mutational signatures were extracted using SigProfilerExtracter. HLA typing was performed with POLYSOLVER and neoantigen prediction performed using pVac-Seq.

For manuscripts utilizing custom algorithms or software that are central to the research but not yet described in published literature, software must be made available to editors and reviewers. We strongly encourage code deposition in a community repository (e.g. GitHub). See the Nature Portfolio guidelines for submitting code & software for further information.

Data

Policy information about availability of data

All manuscripts must include a data availability statement. This statement should provide the following information, where applicable:

- Accession codes, unique identifiers, or web links for publicly available datasets
- A description of any restrictions on data availability
- For clinical datasets or third party data, please ensure that the statement adheres to our policy

The data supporting the findings of this study are available within the Genomics England Research Environment, a secure cloud workspace. An example for details

on how to access data for this publication can be found at https://re-docs.genomicsengland.co.uk/pan_cancer_pub/. Additional processed aggregated data supporting the findings presented in this manuscript can be found in the Supplementary Tables. To access genomic and clinical data within this Research Environment, researchers must first apply to become a member of either the Genomics England Research Network (https://www.genomicsengland.co.uk/research/academic) or the Discovery Forum (industry partners https://www.genomicsengland.co.uk/research/research-environment). The process for joining the network is described at https://www.genomicsengland.co.uk/research/academic/join-gecip and consists of the following steps:
Your institution will need to sign a participation agreement available at https://files.genomicsengland.co.uk/documents/Genomics-England-GeCIP-Participation-Agreement-v2.0.pdf and email the signed version to gecip-help@genomicsengland.co.uk.
Once you have confirmed your institution is registered and have found a domain of interest, you can apply through the online form at https://www.genomicsengland.co.uk/research/academic/join-gecip. Once your Research Portal account is created you will be able to login and track your application.
Your application will be reviewed within 10 working days.
Your institution will validate your affiliation.
You will complete online Information Governance training and will be granted access to the Research Environment within 2 days of passing the online training.
Data that has been made available to registered users include: alignments in BAM or CRAM format, annotated variant calls in VCF format, signatures assignment, tumour mutation burden, sequencing quality metrics, summary of findings that is shared with Genomic Lab Hubs, secondary clinical data as described in this paper. Further details of the types of data available (for example, mortality, hospital episode statistics and treatment data) can be found at https://re-docs.genomicsengland.co.uk/data_overview/. Germline variants can be explored in Interactive Variant Analysis Browser (see description at https://re-docs.genomicsengland.co.uk/iva_variant/). Cancer patients cohort and longitudinal clinical information on treatment and mortality can be explored with Participant Explorer (see description at https://re-docs.genomicsengland.co.uk/pxa/).

Research involving human participants, their data, or biological material

Policy information about studies with human participants or human data. See also policy information about sex, gender (identity/presentation), and sexual orientation and race, ethnicity and racism.

Reporting on sex and gender	Patient sex was determined by generating XX or XY karyotypes from germline WGS data.
Reporting on race, ethnicity, or other socially relevant groupings	We outline the frequency of self reported ethnicity to highlight no significant difference between our two cohorts.
Population characteristics	Age, tumour grade, hormone-receptor status, nodal disease status
Recruitment	Details of recruitment are given by Genomics England - https://www.genomicsengland.co.uk/initiatives/100000-genomes-project/documentation
Ethics oversight	Details provided by Genomics England - https://www.genomicsengland.co.uk/initiatives/100000-genomes-project/documentation

Note that full information on the approval of the study protocol must also be provided in the manuscript.

Field-specific reporting

Please select the one below that is the best fit for your research. If you are not sure, read the appropriate sections before making your selection.

☒ Life sciences ☐ Behavioural & social sciences ☐ Ecological, evolutionary & environmental sciences

For a reference copy of the document with all sections, see nature.com/documents/nr-reporting-summary-flat.pdf

Life sciences study design

All studies must disclose on these points even when the disclosure is negative.

Sample size	Details of sample size are reported in the manuscript and supplementary methods.
Data exclusions	Data exclusions are reported in the supplementary methods.
Replication	Replication was performed by comparison with previously reported studies. Our data was processed and analysed with freely available software allowing for replication.
Randomization	Replication was performed by comparison with previously reported studies. Our data was processed and analysed with freely available software allowing for replication.
Blinding	No blinding applied.

Reporting for specific materials, systems and methods

We require information from authors about some types of materials, experimental systems and methods used in many studies. Here, indicate whether each material, system or method listed is relevant to your study. If you are not sure if a list item applies to your research, read the appropriate section before selecting a response.

Materials & experimental systems

n/a	Involved in the study
☒	☐ Antibodies
☒	☐ Eukaryotic cell lines
☒	☐ Palaeontology and archaeology
☒	☐ Animals and other organisms
☐	☒ Clinical data
☒	☐ Dual use research of concern
☒	☐ Plants

Methods

n/a	Involved in the study
☒	☐ ChIP-seq
☒	☐ Flow cytometry
☒	☐ MRI-based neuroimaging

Clinical data

Policy information about clinical studies
All manuscripts should comply with the ICMJE guidelines for publication of clinical research and a completed CONSORT checklist must be included with all submissions.

Clinical trial registration	N/A
Study protocol	The study protocol is provided by Genomics England - https://www.genomicsengland.co.uk/initiatives/100000-genomes-project/documentation
Data collection	Data collection was carried out by Genomic England - https://www.genomicsengland.co.uk/initiatives/100000-genomes-project/documentation
Outcomes	Outcomes were adjusted and unadjusted correlations between clinical and molecular features.

Plants

Seed stocks	*Report on the source of all seed stocks or other plant material used. If applicable, state the seed stock centre and catalogue number. If plant specimens were collected from the field, describe the collection location, date and sampling procedures.*
Novel plant genotypes	*Describe the methods by which all novel plant genotypes were produced. This includes those generated by transgenic approaches, gene editing, chemical/radiation-based mutagenesis and hybridization. For transgenic lines, describe the transformation method, the number of independent lines analyzed and the generation upon which experiments were performed. For gene-edited lines, describe the editor used, the endogenous sequence targeted for editing, the targeting guide RNA sequence (if applicable) and how the editor was applied.*
Authentication	*Describe any authentication procedures for each seed stock used or novel genotype generated. Describe any experiments used to assess the effect of a mutation and, where applicable, how potential secondary effects (e.g. second site T-DNA insertions, mosiacism, off-target gene editing) were examined.*
